# Case report: *LAMC3-*associated cortical malformations: Case report of a novel stop-gain variant and literature review

**DOI:** 10.3389/fgene.2022.990350

**Published:** 2023-01-06

**Authors:** Giovanni Falcicchio, Antonella Riva, Angela La Neve, Michele Iacomino, Patrizia Lastella, Patrizia Suppressa, Vittorio Sciruicchio, Maria Trojano, Pasquale Striano

**Affiliations:** ^1^ Department of Basic Medical Sciences, Neurosciences and Sense Organs, University of Bari, Bari, Italy; ^2^ Paediatric Neurology and Muscular Diseases Unit, IRCCS Istituto Giannina Gaslini, Genoa, Italy; ^3^ Department of Neurosciences, Rehabilitation, Ophthalmology, Genetics, Maternal and Child Health, University of Genoa, Genoa, Italy; ^4^ Unit of Medical Genetics, IRCCS Istituto Giannina Gaslini, Genoa, Italy; ^5^ Department of Internal Medicine and Rare Diseases Centre “C. Frugoni”, University Hospital of Bari, Bari, Italy; ^6^ Children Epilepsy and EEG Centre, San Paolo Hospital, Bari, Italy

**Keywords:** cortical malformations, epilepsy, exome sequencing, genetic mutations, *LAMC3* case report

## Abstract

**Background:** Malformations of cortical development (MCDs) can lead to peculiar neuroradiological patterns and clinical presentations (i.e., seizures, cerebral palsy, and intellectual disability) according to the specific genetic pathway of the brain development involved; and yet a certain degree of phenotypic heterogeneity exists even when the same gene is affected. Here we report a man with an malformations of cortical development extending beyond occipital lobes associated with a novel stop-gain variant in *LAMC3*.

**Case presentation:** The patient is a 28-year-old man suffering from drug-resistant epilepsy and moderate intellectual disability. He underwent a brain magnetic resonance imaging showing polymicrogyria involving occipital and temporal lobes bilaterally. After performing exome sequencing, a novel stop-gain variant in *LAMC3* (c.3871C>T; p. Arg1291*) was identified. According to the cortical alteration of the temporal regions, temporal seizures were detected; instead, the patient did not report occipital seizures. Different pharmacological and non-pharmacological interventions (*i.e.,* vagus nerve stimulation) were unsuccessful, even though a partial seizure reduction was obtained after cenobamate administration.

**Conclusion:** Our case report confirms that variants of a gene known to be related to specific clinical and neuroradiological pictures can unexpectedly lead to new phenotypes involving different areas of the brain.

## Background

Malformations of cortical development (MCDs) are a heterogeneous group of abnormal cerebral cortex formation disorders affecting people from early childhood to early adulthood (Severino et al., 2020). The most common clinical presentations of MCDs are epilepsy and/or neurodevelopmental delay (Barkovich et al., 2012). Knowledge about MCDs has greatly increased over the years, leading to the classification of MCDs based on neuroradiological patterns (Severino et al., 2020). Moreover, thanks to the evolution of molecular biology and genetics, more than 100 genes have been identified and associated with different types of MCDs (Kuzniecky, 2015; Accogli et al., 2020). Recessive *LAMC3* gene variants cause occipital cortical malformations (Barak et al., 2011), such as pachygyria or polymicrogyria. However, the spectrum of *LAMC3*-associated cortical malformations has progressively expanded over time, showing that other cortical areas could be affected and that occipital lobes could even be spared in some cases (Zambonin et al., 2018; Kasper et al., 2020; De Angelis et al., 2021).

We report the clinical and neuroradiological features of a patient with a novel homozygous stop-gain variant in *LAMC3* (c.3871C>T; p. Arg1291*) identified through whole exome sequencing (WES).

### Case presentation

**FIGURE 1:**
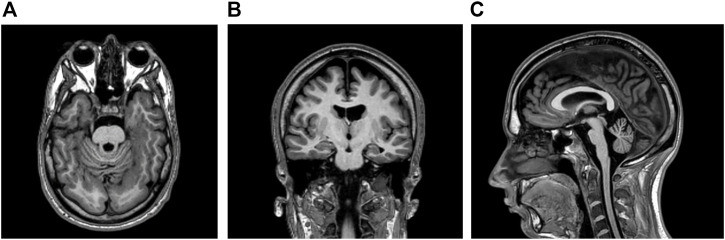
**(A,B)** Brain magnetic resonance imaging (MRI) showing bilateral polymicrogyria in temporal and occipital lobes (T1-weighted axial and coronal sequences respectively) **(C)** Atrophy in pons and cerebellum was detected in the T1-weighted sagittal sequence.

This right-handed, 28-year-old Caucasian man had an unremarkable antenatal and perinatal history. His parents were non-consanguineous and healthy. His older sister did not have known medical conditions. No familial history of genetic, metabolic, or neurological diseases was reported. Educational support was necessary during school years. At the age of six, the patient started to experience daily seizures—often several times a day - with different semeiology: 1) episodes of symmetrical myoclonic spasms of the upper limbs; 2) involuntary movements of both eyes in different directions, followed by behavioural arrest, and impaired awareness with unilateral/bilateral manual automatisms (i.e., manipulating, nose-wiping, and hair-fixing). Multiple antiseizure medications (i.e., lamotrigine, phenobarbital, nitrazepam, vigabatrin, clobazam, topiramate, lacosamide, levetiracetam, oxcarbazepine, brivaracetam) were unsuccessfully administered. The patient came to our attention at the age of 23, when he was taking rufinamide 3,200 mg/day, valproate 1,000 mg/day, and carbamazepine 800 mg/day. Daily seizures with different semeiology (myoclonic, atonic, focal motor, and bilateral tonic-clonic seizures) were reported. Neurological examination was unremarkable except for divergent strabismus in the left eye. No dysmorphic features were identified. Interictal electroencephalogram showed spikes, polyspikes, and polyspike-and-waves localized in the occipital regions. Brain magnetic resonance imaging (MRI) was also performed revealing bilateral temporal and occipital polymicrogyria; atrophy of the pons and cerebellum was also detected (Figure 1). Neuropsychological assessment revealed moderate intellectual disability (ID) with a low intelligence quotient (I.Q. = 48).

Vagus nerve stimulation was implanted, without substantial variation neither in seizure intensity nor in frequency. Oral immunotherapy (prednisone) was ineffective as well. Finally, cenobamate was administered and a partial reduction in seizure frequency was registered (from daily to weekly). At the last follow-up, antiseizure treatment included valproate 1,500 mg/day, zonisamide 400 mg/day, and cenobamate 300 mg/day.

### Genetic analysis

WES was conducted by Research & Innovation Genetics in Padua, Italy. Proband’s and parents’ genomic DNA was extracted from peripheral blood. The DNA library was constructed using a SureSelect All Exon 6 (Agilent) kit. Whole Exome sequencing runs were performed on the NovaSeq6000 platform (Illumina Inc., San Diego, CA, United States) with 150-base paired-end reads. Paired-end reads were aligned to the reference human genome sequence (GRCh37/hg19) using BWA algorithm (Li and Durbin, 2009). Sequences without specific alignment were excluded. The bioinformatic analysis was performed by GATK, samtools and bcftools packages. Mean sequencing depth of 100× and 99.04% of targeted regions covered at 20× depth were obtained. 273 genes already related to MCDs and epilepsy of suspected genetic etiology were prioritized in the analysis (Supplementary Table S1). Population databases (i.e., The Exome Aggregation Consortium, 1000 Genomes Project, GnomAD), genetic clinical databases (i.e., OMIM, ClinVar, HGMD) and *in silico* tools (Polyphen2, Sift, CADD, Mutation Taster, Mutation Assessor) were used to screen the variant. A new very rare stop-gain variant c.3871C>T (p.Arg1291*) in *LAMC3* was identified. It is a loss-of-function variant, and it is predicted to be pathogenic according to ACMG criteria (PVS1,PM2,PP5) (Richards et al., 2015). Sanger sequencing was performed to validate the heterozygous state in the unaffected parents.

## Discussion and conclusions

*LAMC3* gene is located on chromosome 9 and encodes the ɣ3 chain of the extracellular laminin family proteins, associated with cell differentiation, migration, and adhesion (Hamill et al., 2009), and it is involved in the organization of the cerebral cortex and its gyration (Radner et al., 2013). Homozygous or compound heterozygous variants in *LAMC3* have been described to cause occipital cortical malformations (OMIM #614115) (Barak et al., 2011). So far, eight unrelated individuals with *LAMC3* gene variants have been identified worldwide and their clinical, genetic, and neuroradiological characteristics are summarized in Table 1. In nearly all reported cases a frameshift or non-sense LAMC3 variant was detected, except for missense variants in compound heterozygosity in two patients (Barak et al., 2011; De Angelis et al., 2021). Differently from the early literature reporting that only occipital lobes were involved in the cortical malformation process (Barak et al., 2011; Afawi et al., 2016), more recent studies (Zambonin et al., 2018; Kasper et al., 2020; De Angelis et al., 2021; Qian et al., 2021) and the structural analysis of previously reported individuals (Urgen et al., 2019) have demonstrated that *LAMC3* variants can cause structural cerebral anomalies going beyond the occipital lobes, which sometimes can even be spared (Kasper et al., 2020; Qian et al., 2021). Awafi et al. identified a consanguineous Arab family in which, out of 14 siblings, all the affected children (n = 6) and one unaffected sibling shared the same *LAMC3* homozygous variant; all the affected family members had epilepsy and ID, but their computed tomography scans were unremarkable. Only one of the affected siblings underwent brain MRI, showing occipital dysplasia. In our patient, polymicrogyria was more extended, involving occipital as well temporal lobes.

**TABLE 1:** Summary of previous LAMC3 variations with associated clinical and neuroradiological characteristics.

Patient	References	Consanguinity	*LAMC3* variation§	ClinVar ID	MRI findings	Clinical picture
#1	Barak et al., (2011)	Yes	c.903_904del (p.Cys301*)	Variation ID: 30416	Bilateral occipital pachygyria and parietal-occipital polymicrogyria	Absence seizures
						Mild developmental delay
#2	Barak et al., (2011)	Yes	c.470G>A (p.Trp157*)	Variation ID: 30417	Bilateral occipital pachygyria and polymicrogyria	Staring and blinking spells
						Average intelligence
#3	Barak et al., (2011)	Yes	c.1156C>T (p.Gln386*)	Variation ID: 30418	Bilateral occipital pachygyria and polymicrogyria	Occipital lobe seizures
			c.1048G>A (p.Gly350Arg)	Variation ID: 30419		Unknown mental development
#4	Afawi et al., (2016)	Yes	c.4086delA (p.Asp1363Thrfs*54)	Variation ID: n/a	Occipital dysplasia	Myoclonic atonic seizures
						Developmental delay
#5	Zambonin et al., (2018)	Yes	c.3190C>T (p.Gln1064*)	Variation ID: n/a	Bilateral frontal, parietal, temporal, occipital polymicrogyria and pachygyria of occipital lobes	Atypical absences and myoclonic-astatic seizures
						Severe developmental delay
#6	Kasper et al., (2020)	No	c.976 + 1G>C	Variation ID: 810444	Bilateral frontal polymicrogyria	Déjà vu, impaired awareness, automatisms
						Normal development
#7	De Angelis et al., (2021)	no	c.1066C>T (p.Arg356Cys)	Variation ID: 691960	Posterior periventricular nodular heterotopia	Voluntary termination of pregnancy
			c.2726A>G (p.Gln909Arg)	Variation ID: 691961		Dysmorphic features
#8	Qian et al., (2021)	no	c.470G>A (p.Trp157*)	Variation ID: 30417	Normal	Absence and tonic-clonic seizures
			c.4030C + 1G>A	Variation ID: n/a		Normal development
#9	Our case	no	c.3871C>T (p.Arg1291*)	Variation ID: 1069700	Bilateral temporal and occipital polymicrogyria	Temporal, myoclonic, generalized and atonic seizures
					Atrophy in pons and cerebellum	Moderate developmental delay

^a^ Nucleotide and amino acid changes are reported.

Polymicrogyria is a heterogeneous MCD which can also be caused by non-genetic factors, with *in utero* infections, prenatal ischemia, or exposure to medications during gestation as the most frequent alternative aetiologies (Raybaud and Widjaja, 2011). The onset of the neurological and cognitive disturbances in the affected patients is between the age of 2 and 13 years and it is often associated with developmental or cognitive impairment (Barak et al., 2011; Afawi et al., 2016; Zambonin et al., 2018), as in our patient. MCDs caused by variants in a single recessive gene demonstrate how a single gene can influence the development of the cortical structure, as well as the phenotype of the individual carrying the variant with different neurological (epilepsy, cerebral palsy) and cognitive (ID) outcomes. Different seizure types (absences, myoclonic-astatic, tonic-clonic, and occipital lobe seizures) have been reported in patients with epilepsy related to LAMC3 variants (Table 1). In our patient, the involvement of the temporal regions was supported by the suggestive seizure semiology indicating onset in the temporal lobe (i.e., behavioural arrest and impaired awareness and oral and manual automatisms).

In summary, we report a novel *LAMC3* variant and broadened the phenotypic spectrum of *LAMC3*-related MCDs showing involvement outside of the occipital area. The identified, likely pathogenic variant is novel and may result in a loss-of-function mechanism, eventually preventing the formation of the related protein.

## Data Availability

The datasets for this article are not publicly available due to concerns regarding participant/patient anonymity. Requests to access the datasets should be directed to the corresponding author.

## Abbreviations

ACMG, American College of Medical Genetics and Genomics; ID, intellectual disability; IQ, intelligence quotient. MCD, malformation of cortical development. MRI, magnetic resonance imaging.NGS, next-generation sequencing; PCR, polymerase chain reaction; WES, whole exome sequencing.
