# Supplementary material for: Case report: LAMC3-associated cortical malformations: Case report of a novel stop-gain variant and literature review
Source: Front Genet. 2023 Jan 6;13:990350. doi: 10.3389/fgene.2022.990350 (PMC9852726; doi:10.3389/fgene.2022.990350)
Supplement: Supplementary file 1 [file DataSheet2.PDF]

AARS	ATR	COL18A1	ETFDH	GPSM2	KIAA2022	NSDHL	POMT2	SERPINI1	SYNJ1
ABAT	B3GALNT2	COL4A1	FARS2	GRIN1	KIF1BP	NTRK2	PPP3CA	SGCE	SZT2
ACTB	B4GAT1	CPA6	FAT4	GRIN2A	KIF2A	NUP133	PPT1	SHH	TBC1D20
ACTG1	BICD2	CPLX1	FGF12	GRIN2B	KIF5C	OCLN	PQBP1	SIK1	TBC1D24
ADAM22	CACNA1A	CRADD	FGFR3	GRN	LAMA2	OFD1	PRDM8	SLC12A5	TCF4
ADGRG1	CAD	CSTB	FKRP	GUF1	LAMB1	ORC1	PRICKLE1	SLC13A5	TMTC3
ADGRV1	CARS2	CTNNA2	FKTN	HCN1	LAMC3	ORC4	PRICKLE2	SLC19A3	TPP1
ADRA2B	CASK	CTNND2	FLNA	HCN2	LARGE1	ORC6	PRIMA1	SLC1A2	TRAK1
ADSL	CCND2	CTSD	FOLR1	HCN4	LGI1	PACS2	PRRT2	SLC2A1	TSC1
AHI1	CDC6	CUL4B	FOXG1	HEPACAM	LIAS	PAFAH1B1	RAB18	SLC25A12	TSC2
AKT1	CDK5RAP2	CUX2	FRRS1L	HIP1	LMNB2	PAX6	RAB39B	SLC25A19	TSEN2
ALDH5A1	CDKL5	CYFIP2	GABBR2	HNRNPU	MCPH1	PCDH19	RAB3GAP1	SLC25A22	TSEN34
ALDH7A1	CDT1	DCHS1	GABRA1	HSD17B4	MDH2	PCNT	RAB3GAP2	SLC35A2	TSEN54
ALG13	CENPJ	DCX	GABRB1	ISPD	MECP2	PHACTR1	RARS2	SLC6A1	TUBA1A
AMT	CEP152	DENND5A	GABRB2	ITPA	MEF2C	PIGA	RELN	SLC6A8	TUBA8
AP3B2	CERS1	DEPDC5	GABRB3	KCNA2	MFSD8	PIGP	RHOBTB2	SLC6A9	TUBB
APC2	CHD2	DNAJC5	GABRG2	KCNB1	MTOR	PIGQ	RIN2	SLC9A6	TUBB2A
ARFGEF2	CHRNA2	DNM1	GAL	KCNC1	NBEA	PIK3R2	RTTN	SMC1A	TUBB2B
ARHGEF9	CHRNA4	DOCK7	GATM	KCNH5	NDE1	PLCB1	RXYLT1	SNAP29	TUBB3
ARV1	CHRNB2	DYNC1H1	GCSH	KCNMA1	NECAP1	PLPBP	SCARB2	SNX27	TUBB4A
ARX	CLN3	EEF1A2	GLDC	KCNQ2	NEDD4L	PNKP	SCN1A	SPTAN1	TUBG1
ASNS	CLN5	EML1	GLS	KCNQ3	NEUROD2	PNPO	SCN1B	SRD5A3	UBA5
ASPM	CLN6	EOMES	GMPPB	KCNQ5	NHLRC1	POLG	SCN2A	SRPX2	UBE3A
ATP1A2	CLN8	EPM2A	GNAO1	KCNT1	NPRL2	POMGNT1	SCN3A	ST3GAL3	VLDLR
ATP1A3	CNPY3	ERMARD	GNAQ	KCNT2	NPRL3	POMGNT2	SCN5A	STIL	WDR45
ATP6V0A2	CNTN2	ETFA	GOSR2	KCTD7	NRXN1	POMK	SCN8A	STRADA	WDR62
ATP6V1A	CNTNAP2	ETFB	GPC3	KDM5C	NSD1	POMT1	SCN9A	STX1B	WWOX
							SEPSECS	STXBP1	YWHAG

Supplementary Table . List of the 273 genes related to MCDs and epilepsy of suspected genetic etiology prioritized in the genetic analysis.
