# Supplementary material for: Case report: LAMC3-associated cortical malformations: Case report of a novel stop-gain variant and literature review
Source: Front Genet. 2023 Jan 6;13:990350. doi: 10.3389/fgene.2022.990350 (PMC9852726; doi:10.3389/fgene.2022.990350)
Supplement: Supplementary file 2 [file DataSheet1.PDF]

2013 CARE Checklist

1. **Title** – The diagnosis or intervention of primary focus followed by the words "case report":

   LAMC3-associated cortical malformations: case report of a novel stop variant and literature review

2. **Key Words** – 2 to 5 key words that identify diagnoses or interventions in this case report (including "case report"):

   Cortical malformations - epilepsy – exome sequencing - genetic mutations – LAMC3 case report

3. **Abstract** – (structured or unstructured)

   - Introduction – What is unique about this case and what does it add to the scientific literature? *This is the first report of a patient carrying the LAMC3 (c.3871C>T; p.Arg1291*) variant.*
   - The patient's main concerns and important clinical findings. *See section "abstract" – "case presentation"*
   - The primary diagnoses, interventions, and outcomes See *section "abstract"- "case presentation"*
   - Conclusion – What are one or more "take-away" lessons from this case report? *Mutations of a gene usually related to a well-known clinical and neuroradiological picture can be unexpectedly associated with different scenarios, including the involvement of different brains areas.*

4. **Introduction** – Briefly summarizes why this case is unique and may include medical literature references:

   In this manuscript, we report a novel homozygous mutation of LAMC3 gene, comparing our findings with previous reports. We underline how the mutation of a single gene can cause a spectrum of variable clinical manifestations and neuroradiological features. Considering the growing importance of genetics in the epilepsy field, it is important to share the different phenotypes a single mutation can give. So far, this is the 9th documented case of LAMC3 mutation worldwide.

   References: *- Kuzniecky R. Epilepsy and malformations of cortical development: new developments. Curr Opin Neurol. 2015 Apr;28(2):151-7. doi: 10.1097/WCO.0000000000000175. PMID: 25695135.*

   - Barak T, Kwan KY, Louvi A, Demirbilek V, Saygı S, Tüysüz B, Choi M, Boyacı H, Doerschner K, Zhu Y, Kaymakçalan H, Yılmaz S, Bakırcıoğlu M, Cağlayan AO, Oztürk AK, Yasuno K, Brunken WJ, Atalar E, Yalçınkaya C, Dinçer A, Bronen RA, Mane S, Ozçelik T, Lifton RP, Sestan N, Bilgüvar K, Günel M. Recessive LAMC3 mutations cause malformations of occipital cortical development. Nat

Genet. 2011 Jun;43(6):590-4. doi: 10.1038/ng.836. Epub 2011 May 15. PMID: 21572413; PMCID: PMC3329933.

- Zambonin JL, Dyment DA, Xi Y, Lamont RE, Hartley T, Miller E, Kerr M; Care4Rare Canada Consortium, Boycott KM, Parboosingh JS, Venkateswaran S. A novel mutation in LAMC3 associated with generalized polymicrogyria of the cortex and epilepsy. Neurogenetics. 2018 Jan;19(1):61-65. doi: 10.1007/s10048-017-0534-4. Epub 2017 Dec 15. PMID: 29247375.

5. **Patient Information**
   - De-identified patient specific information. *Section "Case presentation"- "Clinical features"*
   - Primary concerns and symptoms of the patient. *Section "Case presentation"- "Clinical features"* (drug-resistant epilepsy, intellectual disability)
   - Medical, family, and psychosocial history including relevant genetic information. *Section "Case presentation"- "Clinical features"* (unremarkable antenatal and perinatal history. His parents and sister are in good health. No familial history of genetic, metabolic, or neurologic disorders. Anti-seizure treatment is reported).
   - Relevant past interventions and their outcomes. *Section "Case presentation"- "Clinical features"* (the lack of response to previous anti-seizure medications and VNS is evidenced).
6. **Clinical Findings** – Describe significant physical examination (PE) and important clinical findings. Section "Case presentation"- "Clinical features" (Neurological examination was unremarkable except for divergent strabismus in the left eye. No dysmorphic features were identified. The neuropsychological assessment revealed moderate intellectual disability (ID) with reduced intelligence quotient (I.Q.=48))
7. **Timeline** – Historical and current information from this episode of care organized as a timeline (figure or table).

Time 0	First neurological examination
Time 1	EEG and MRI
Time 2	Genetic testing
Time 3	Anti-seizure treatment

8. **Diagnostic Assessment**

- Diagnostic methods (PE, laboratory testing, imaging, surveys). *See Section "Case presentation -clinical features"* (Interictal electroencephalogram showed spikes, polyspike, and polyspike-and-waves in the occipital regions. Brain magnetic resonance imaging (MRI) was also performed revealing bilateral temporal and occipital cortical malformation, identified as polymicrogyria; atrophy of the pons and cerebellum was also detected)
- Diagnostic challenges. *See section "Mutation Identification" (considering the intellectual disability, the refractory epilepsy and that pachygyria involved occipital regions of the brain, whole-exome sequencing was performed to confirm/exclude genetic etiology of the problem)*
- Diagnosis (including other diagnoses considered). *See section "Mutation identification"* (a new stop variant c.3871C>T (p.Arg1291*) in the LAMC3 gene was identified)
- Prognostic characteristics when applicable. N/A

9. **Therapeutic Intervention**

- Types of therapeutic intervention (pharmacologic, surgical, preventive). *See Section "Case presentation"- "Clinical features"*
- Administration of therapeutic intervention (dosage, strength, duration). *See Section "Case presentation"- "Clinical features"*

10. Changes in therapeutic interventions with explanations. See *Section "Case presentation"- "Clinical features"*

11. **Follow-up and Outcomes**

- Clinician- and patient-assessed outcomes if available. *Section "Case presentation"- "Clinical features"*
- Important follow-up diagnostic and other test results. *Section "Case presentation"- "Clinical features"*
- Intervention adherence and tolerability. (How was this assessed?) N/A
- Adverse and unanticipated events. N/A

12. **Discussion**

- Strengths and limitations in your approach to this case. *Here we reported a novel LAMC3 variant and broadened the phenotypic spectrum of LAMC3-related MCDs clearly showing involvement outside of the occipital area.*

- Discussion of the relevant medical literature. *See section Discussion and conclusions (analogies and differences with the previous literature are reported)*
- The rationale for your conclusions. *the variant we identified has not been reported in the previous literature and can be considered likely pathogenic because, due to a loss-of-function mechanism, it prevents the formation of the protein*

13. The primary "take-away" lessons from this case report (without references) in a one paragraph conclusion. *The spectrum of LAMC3-associated cortical malformations has widened over time, showing that other cortical areas could be involved, and, in some cases, the occipital lobes could even be spared.*
14. **Patient Perspective** – The patient should share their perspective on the treatment(s) they received. N/A
15. **Informed Consent** – The patient should give informed consent. (Provide if requested.) *Written informed consent has been obtained.*
